# Fiber Formation and Structural Development of HBA/HNA Thermotropic Liquid Crystalline Polymer in High-Speed Melt Spinning

**DOI:** 10.3390/polym13071134

**Published:** 2021-04-02

**Authors:** Bo Seok Song, Jun Young Lee, Sun Hwa Jang, Wan-Gyu Hahm

**Affiliations:** 1 School of Chemical Engineering, Sungkyunkwan University (SKKU), Suwon 16419, Korea; bseok91@gmail.com; 2 Advanced Textile R&D Department, Korea Institute of Industrial Technology (KITECH), Ansan 15588, Korea; 3 Business Development Team, SEYANG POLYMER Co., Ltd., Anseong 17602, Korea; shjang@seyangpolymer.com

**Keywords:** thermotropic liquid crystalline polymer (TLCP), melt spinning, fiber formation, structure development, shear rate, draft, X-ray analysis

## Abstract

High-speed melt spinning of thermotropic liquid crystalline polymer (TLCP) resin composed of 4-hydroxybenzoic acid (HBA) and 2-hydroxy-6-napthoic acid (HNA) monomers in a molar ratio of 73/27 was conducted to investigate the characteristic structure development of the fibers under industrial spinning conditions, and the obtained as-spun TLCP fibers were analyzed in detail. The tensile strength and modulus of the fibers increased with shear rate in nozzle hole, draft in spin-line and spinning temperature and exhibited the high values of approximately 1.1 and 63 GPa, respectively, comparable to those of industrial as-spun TLCP fibers, at a shear rate of 70,000 s^−1^ and a draft of 25. X-ray diffraction demonstrated that the mechanical properties of the fibers increased with the crystalline orientation factor (*f_c_*) and the fractions of highly oriented crystalline and non-crystalline anisotropic phases. The results of structure analysis indicated that a characteristic skin-core structure developed at high drafts (i.e., spinning velocity) and low spinning temperatures, which contributed to weakening the mechanical properties of the TLCP fibers. It is supposed that this heterogeneous structure in the cross-section of the fibers was induced by differences in the cooling rates of the skin and core of the fiber in the spin-line.

## 1. Introduction

Thermotropic liquid crystalline polymers (TLCPs), which exhibit liquid crystal behavior at adequate temperature, is one of the typical anisotropic polymers and represented almost exclusively by aromatic copolyesters [1,2,3]. Especially, TLCP resins such as the copolymers, composed of 4-hydroxybenzoic acid (HBA) and 2-hydroxy-6-napthoic acid (HNA) in a molar ratio of 73/27, has attracted attention as one of the commercially available resins, because it possesses excellent melt processability to manufacture technical fibers, films and injected parts through the conventional melt processes [4,5,6,7].

In the case of melt spinning for TLCP resins, liquid crystal domains in the polymer melt can be readily oriented in the direction of the fiber axis by the shear and elongational forces, and the resultant TLCP fibers exhibit excellent mechanical properties, similar to those of conventional lyotropic LCP fibers such as aramid fibers [8], with better resistance to moisture, chemicals and abrasion [9]. Thus, TLCP fibers have been used as high-performance materials in the special industrial applications such as aerospace, construction and marine fields, and many studies have been conducted to enhance the properties and productivity of TLCP fibers.

Kenig [10] performed the experimental and modeling study on the orientation development characteristics of HBA/HNA TLCP under the shear rates of 1000~10,000 s^−1^ and the draft (i.e., drawdown ratio) of 2~10 using a capillary rheometer and reported that the TLCP that exhibited higher orientability in elongational flow also exhibited higher orientability in shear flow. Cuculo and Chen [11] investigated the flow behavior and the effect of the spinning conditions on the fiber properties and structure of poly(ethylene terephthalate) modified with 60 mol% p-hydroxybenzoic acid (PET/60PHB) and suggested that the mechanical property of as-spun fibers could be improved when a high extrusion rate and/or a high draft was used. Muramatsu and Krigbaum [12] also conducted rheological measurements and fiber spinning for HBA/HNA (58/42 molar ratio) under the shear rates of 1~10,000 s^−1^ and the draft of 1~15 using a capillary rheometer and reported that the mechanical property of as-spun fibers tended to increase as the draft and spinning temperature increased. Wissbrun and Ide [13,14,15,16] reported that HBA/HNA TLCP can be processed at reduced temperatures by controlling the thermal history to improve their mechanical properties, and thus, the control of elongational flow of the TLCP melt using small orifices during fiber spinning can contribute to enhance molecular orientation and improve product properties.

The results of these prior studies indicate that aromatic copolyester TLCP resins exhibit high shear thinning behavior in the nozzle hole, and its fiber formation and structure development in the spin-line complete immediately after extruding from the nozzle hole. These mean that the fiber formation and structure development of the TLCP fibers are considerably influenced by the spinning conditions such as shear rate in nozzle hole, draft in spin-line and spinning temperature. However, few studies have explored how the structure and mechanical property of the TLCP fibers are affected by the melt spinning conditions on an extreme industrial scale. 

In this study, we conducted high-speed melt spinning of HBA/HNA TLCP resin under industrial spinning conditions, the shear rates of 30,000~70,000 s^−1^ and the draft of 10~25 using a pilot-scale melt spinning process and investigated the correlation between the structural and mechanical properties of the as-spun TLCP fibers by tensile testing, field emission scanning electron microscopy (FESEM) and two-dimensional wide-angle X-ray diffraction (2D-WAXD). The relationship between the mechanical properties and structure of the fibers was assessed in detail, with a focus on the changes in crystalline orientation factor (*f*_c_) and the volume fractions of oriented crystalline and non-crystalline anisotropic phases in the as-spun TLCP fibers.

## 2. Materials and Methods

### 2.1. Materials

Commercial grade TLCP resin, synthesized with HBA and HNA monomers in a molar ratio of 73/27, for fiber spinning was supplied by Seyang Polymer Co., Ltd., (Anseong, Korea).

### 2.2. Melt Spinning

**Figure 1:**
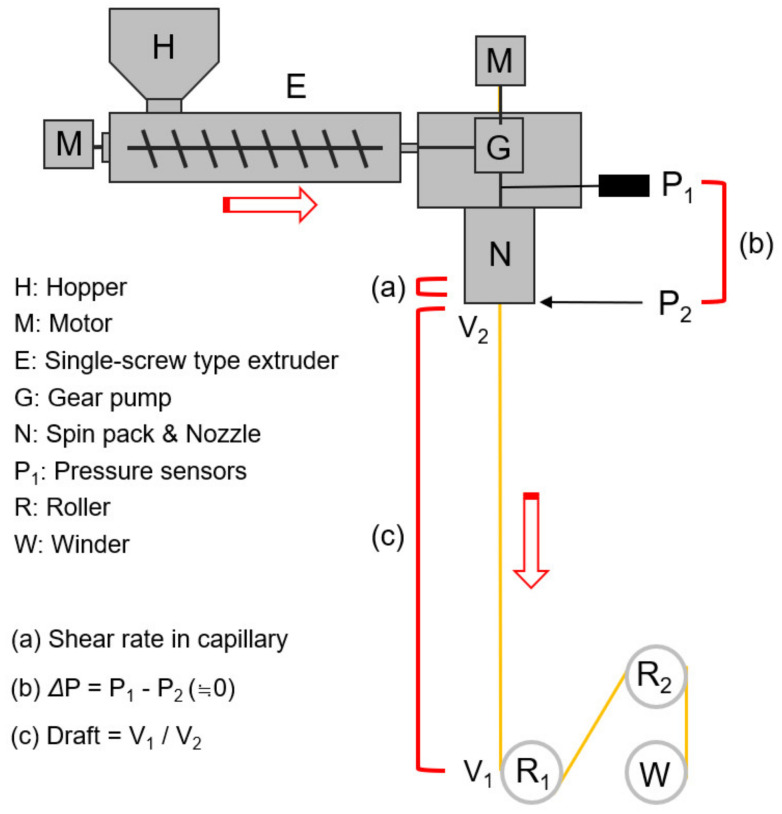
Schematic diagram of high-speed melt spinning process for thermotropic liquid crystalline polymer (TLCP) resin.

Figure 1 shows a schematic diagram of the melt spinning process. The TLCP resin, dried at 120 °C for 12 h to prevent degradation due to hydrolysis during the process, was melted using a single screw type extruder of Ø 25 mm and extruded from a nozzle by a gear pump. A cooling device was not used in the spin-line, and the temperature and relative humidity in the room were controlled within 15~20 °C and 40~60%, respectively.

In this study, to systematically investigate the variations of structure and mechanical property of the TLCP fibers by the spinning conditions, various TLCP as-spun fibers were prepared by varying the throughput rate (0.78~1.81 g·min^−1^·hole^−1^) and take-up velocity (550~2200 m·min^−1^) under two spinning temperatures (285 and 295 °C). The apparent shear rate in the nozzle hole and the draft (i.e., drawdown ratio) in the spin-line were controlled in the range of approximately 30,000~70,000 s^−1^ and 10~25, respectively, comparable to the spinning conditions used in industrial settings.

The apparent shear rate (*γ*_app_) of the TLCP melt in the nozzle hole was calculated using Equation (1) [17], assuming the TLCP melt exhibits an incompressible and Newtonian laminar flow for each flow rate in the nozzle hole, and the spin-line draft was calculated using Equations (2) and (3) [18].

*γ*_app_ = 4*Q*_v_/*πr*_0_^3^, (1)

Draft = *V*_1_/*V*_2_, (2)

*V*_2_ = 4*Q*_m_/*πD*^2^*ρH*_n_, (3)

where *Q*_v_ and *Q*_m_ are the volumetric and mass flow rate of the polymer, respectively; *r*_0_ is the radius of the hole (capillary); *V*_1_ and *V*_2_ are the take-up velocity and throughput rate, respectively; *H*_n_ is the spinneret hole number; *D* is the hole diameter of the spinneret (*r*_0_ = D/2); *ρ* is the melt density (≈1.30) of the TLCP, estimated using a precise metering gear pump in the spinning process.

Table 1 shows the code names of as-spun samples obtained according to the spinning conditions. The samples (L-30K-10 and H-30K-10) at shear rate 30 and draft 10 could not prepared, because the take-up velocity required to obtain the samples was lower than the minimum operating velocity (500 m·min^−1^) of the winder in this study.

### 2.3. FESEM

The surface morphology of the as-spun TLCP fibers was observed using FESEM (SU8010, Hitachi, Tokyo, Japan) with an acceleration voltage of 10 kV after sputter coating with platinum (Pt).

### 2.4. Tensile Test

The tensile properties of the as-spun fibers were measured using a tensile mechanical testing machine for single fibers (FAVIMAT, Textechno, Moenchengladbach, Germany) according to ASTM D3822. The gauge length and crosshead speed were 40 mm and 20 mm·min^−1^, respectively. Twenty tensile test measurements were conducted for each sample, and the average value was reported.

### 2.5. 2D-WAXD

Two-dimensional WAXD analysis of the TLCP as-spun fibers was performed using a RIGAKU D/MAX-2500 R-AXIS apparatus (Rigaku, Tokyo, Japan), for which a nickel-filtered Cu Kα X-ray source (*λ* = 1.541 Å) generated at 50 kV and 100 mA was used. The beam diameter was approximately 0.5 mm, and the camera length and exposure time were 120 mm and 20 min, respectively. The sample holder used for the measurement was a rectangular shape (30 mm length and 20 mm width) with a hole of 5 mm diameter in the center. A bundle of fibers, arranged evenly in the direction of the fiber axis, was prepared with a width of approximately 3 mm in the center of the holder, and the measurement was performed on the fiber sample placed in the center of the holder.

## 3. Results and Discussion

### 3.1. Rheological Properties

**Figure 2:**
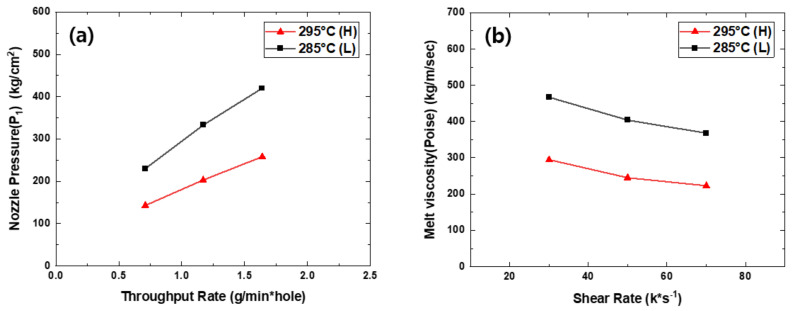
Relationship between (**a**) nozzle pressure (*P*_1_) and throughput rate and between (**b**) melt viscosity (poise) and shear rate for the TLCP resin in the spinning nozzle hole at the different spinning temperatures of 285 and 295 °C.

Figure 2a shows the effect of the throughput rate and spinning temperature on the nozzle pressure in the TLCP spinning process, as measured by a pressure sensor (*P*_1_) positioned between the metering gear pump and the spinning nozzle (Figure 1). As expected, the nozzle pressure increased steeply with the throughput rate and decreased as the spinning temperature increased. If we assume that the pressure (*P*_2_) at the exit of the nozzle hole to be zero and the TLCP melt exhibits an incompressible and Newtonian laminar flow for each *Q*_m_ in the nozzle hole, the apparent melt viscosity (*η*_app_) of the TLCP melt in the nozzle hole of a length *L* can be calculated using Hagen–Poiseuille’s law [17] and written as

*η*_app_ = *πD*^4^(*P*_1_ − *P*_2_)*ρ*/128*Q*_m_*L* (4)

Figure 2b shows the relationship between the apparent melt viscosity (*η*_app_) and apparent shear rate (*γ*_app_) of the TLCP melt in the vicinity of the nozzle hole, calculated from the values of nozzle pressure and throughput rate shown in Figure 2a. When the spinning temperature was 295 °C, the TLCP viscosity was approximately 295 kg/m∙sec at a shear rate of 30,000 s^−1^ and decreased continuously as the shear rate increased to 70,000 s^−1^, indicating that the shear thinning effect due to stretching (i.e., orientation) and disentangling of the TLCP still occurs even at this extremely high shear rate. The melt viscosity was much higher at 285 than at 295 °C but still decreased steeply as the shear rate increased. This suggests that the mobility of the TLCP melt structure was considerably suppressed at the lower spinning temperature. It is expected that the change in melt structure in the vicinity of the nozzle hole owing to changes to the shear rate and spinning temperature will affect the structural development of the fibers in the spinning line and the mechanical properties of the as-spun fibers.

### 3.2. FESEM Analysis

**Figure 3:**
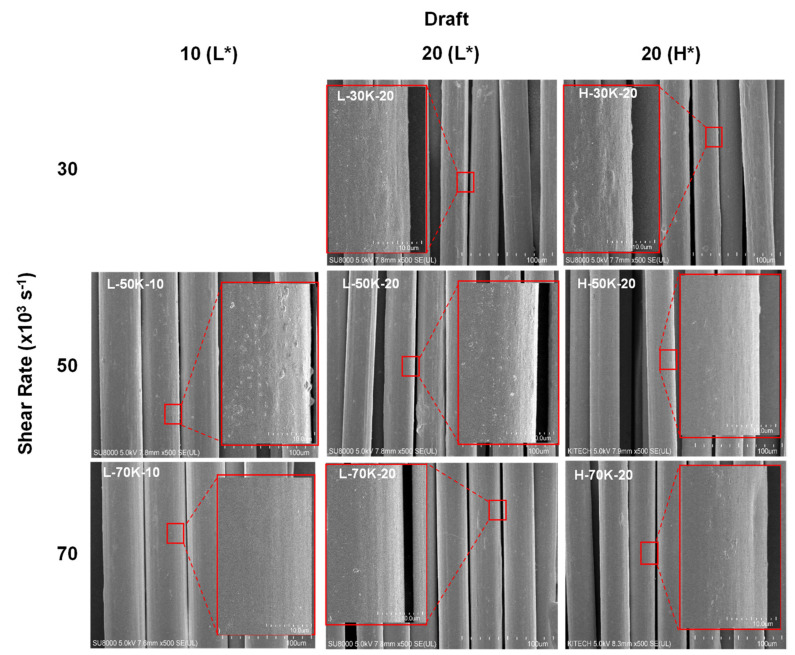
SEM images of the surface of the TLCP as-spun fibers obtained at various spinning conditions. L* and H* indicate the spinning temperatures of 285 and 295 °C, respectively.

Morphological analyses of the TLCP as-spun fibers prepared under different spinning conditions were performed using FESEM (Figure 3). All the as-spun fibers had a uniform fiber shape without any defects such as melt fractures due to high shear rates or irregular pores due to thermal degradation, indicating that the TLCP exhibited stable melt spinnability even under the extreme spinning conditions in this study. When the spinning temperature was 285 °C, the as-spun fibers had a diameter of approximately 48 μm when the draft (i.e., spinning stress) was 10 (L-50K-10 and L-70K-10), which decreased to approximately 30 μm when the draft increased to 20 (L-50K-20 and L-70K-20). The fiber diameters showed negligible changes when the shear rate (i.e., throughput rate) changed, because we simultaneously changed the spinning velocity to maintain the same draft. This allowed us to accurately investigate the effect of shear rate on fiber structure development, while minimizing the effect of draft. At a draft of 20, the diameters of the fibers spun at 295 and 285 °C were similar, despite the change in spinning temperature.

At the low spinning temperature of 285 °C, the TLCP as-spun fibers obtained at shear rates of 30 and 50,000 s^−1^ had a slightly rough surface, which tended to become smoother as the shear rate increased to 70,000 s^−1^. The fiber surface also changed with shear rate when the spinning temperature was 295 °C, but the smoother surface started to appear at a relatively lower shear rate of 50,000 s^−1^. The fiber surface was hardly affected by the draft. These results suggest that the TLCP fiber surface structure was influenced by the shear rate and temperature conditions in the nozzle hole, which could be improved by using a higher shear rate and spinning temperature.

### 3.3. Mechanical Properties

**Figure 4:**
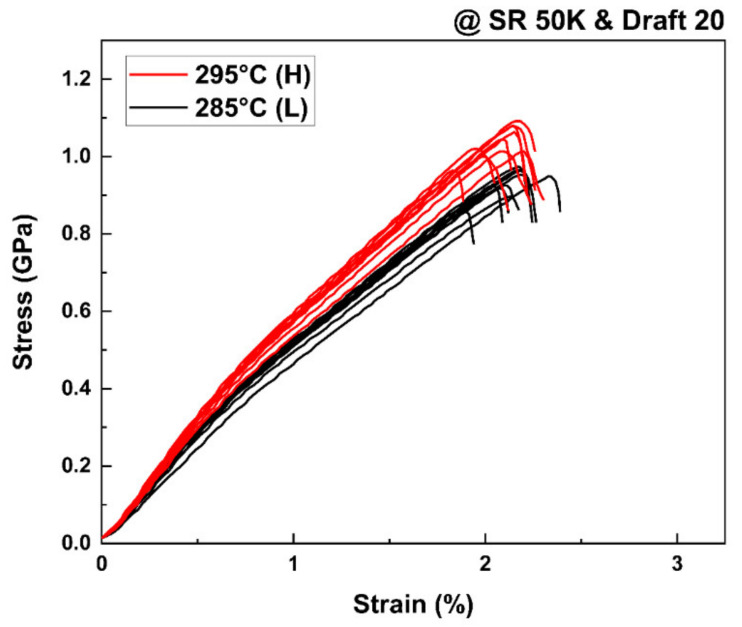
Variation of stress-stain curves of the as-spun TLCP fibers, prepared at the shear rate of 50,000 s^−1^ and the draft of 20, by the change in spinning temperatures (285 and 295 °C).

**Figure 5:**
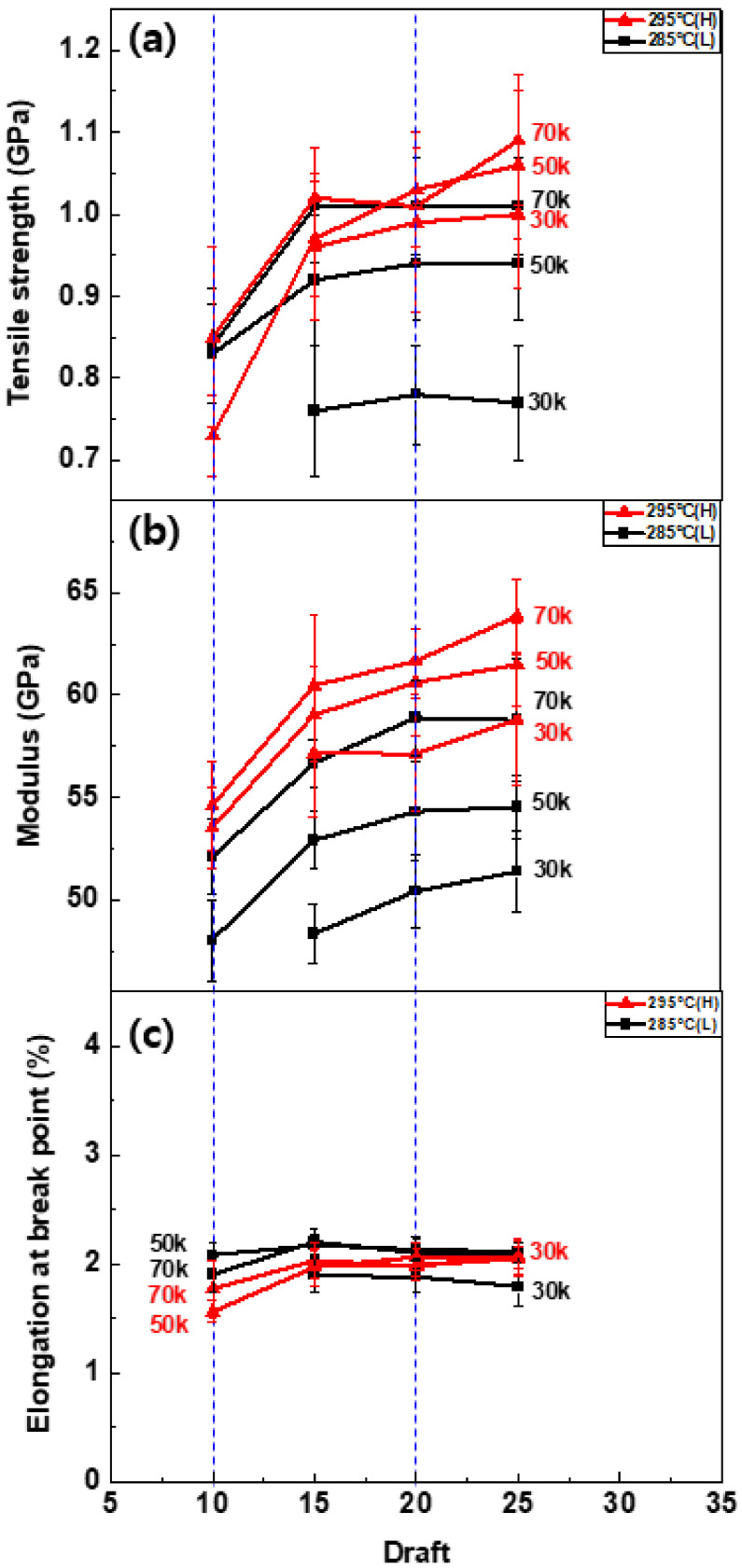
Variations of (**a**) tensile strength, (**b**) modulus and (**c**) elongation at break of as-spun TLCP fibers by the change in the spinning draft. The blue dotted lines indicate the as-spun fibers analyzed by FESEM (Figure 3) and two-dimensional wide-angle X-ray diffraction (2D-WAXD) (Figure 6), respectively.

Figure 4 shows, as an example, stress-stain curves of the TLCP as-spun fibers obtained at a shear rate of 50,000 s^−1^ and draft of 20 under different spinning temperatures in this study. It is observed that the stress increased linearly with strain until the maximum stress was reached at a low strain of approximately 2.0%, suggesting that a highly oriented structure developed in the as-spun fibers during melt spinning. However, the maximum stress (i.e., tensile strength) tended to increase as the spinning temperature increased, which indicates that the degree of structural development in the fibers was affected by the spinning conditions. The characteristic relationship between the mechanical properties of the TLCP as-spun fibers and the spinning conditions is plotted in detail in Figure 5.

At a spinning temperature of 295 °C, the tensile strength (Figure 5a) increased steeply as the draft increased from 10 to 15 and, then, increased slowly or remained similar as the draft increased further. The tensile strength also tended to increase as the shear rate increased. Thus, the highest tensile strength of approximately 1.1 GPa was observed for the fiber obtained under the maximum shear rate (70,000 s^−1^) and draft (25) conditions. The tensile strength of the as-spun fibers obtained at the low spinning temperature of 285 °C showed a similar tendency. However, the tensile strength was much lower at the low shear rate of 30,000 s^−1^, and the curves shifted more obviously toward higher values as the shear rate increased compared with the curves of the fibers obtained at the higher spinning temperature.

These results indicate that the mechanical properties of the TLCP fibers were considerably influenced by the shear rate and draft in the spin-line. In addition, it is speculated that the effects of these conditions could be more enhanced or suppressed by changing the spinning temperature. In other words, considering the relationship between the mechanical properties of fibers and their structural development, it is supposed that the structural development in the spin-line is mainly governed by the temperature-dependent melt structure behavior, and the degree of structural development due to the forces such as the shear rate (i.e., shear stress) and draft (i.e., spin-line stress) can be enhanced by increasing the mobility of the melt structure. It can be further controlled by the spinning temperature as predicted by the rheological properties of the TLCP resin.

The modulus curves (Figure 5b) further revealed the effects of the spinning conditions on the structural development of TLCP fibers. Similar to the tensile strength variation, the modulus of the as-spun fibers also increased with the draft, and the curves shifted upward as the shear rate increased. All the modulus curves of the fibers prepared at the high spinning temperature of 295 °C were higher than those of the fibers prepared at 285 °C, which confirmed that the degree of structural development of the fibers due to the stress can be enhanced significantly by increasing the spinning temperature.

The elongation at the break of the as-spun fibers showed negligible changes depending on the spinning conditions, with low values of approximately 2.0%. This high rigidity and low elongation property of the as-spun fibers appears to be due to the low mobility of characteristic aromatic molecular structures of HBA/HNA in the TLCP [1,2].

### 3.4. 2D-WAXD

**Figure 6:**
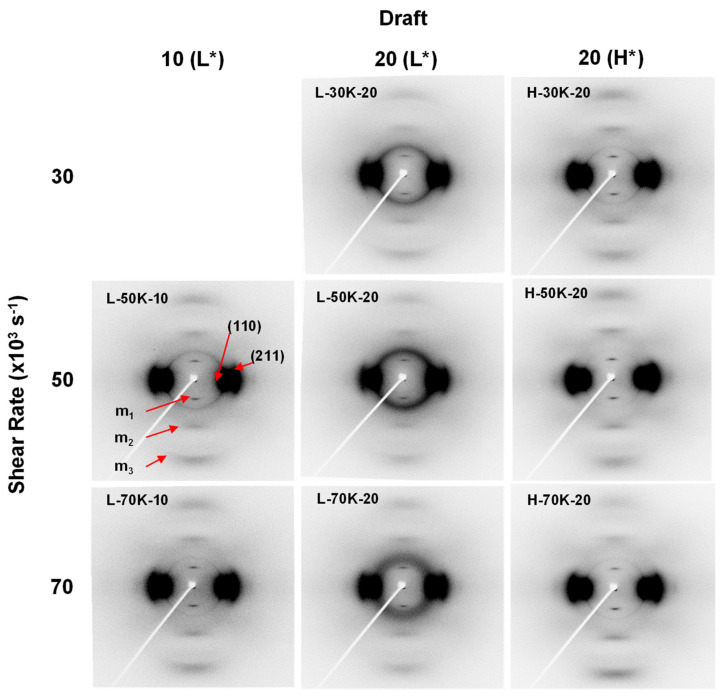
Two-dimensional WAXD patterns of as-spun TLCP fibers obtained at various spinning conditions. L* and H* indicate the spinning temperatures of 285 and 295 °C, respectively.

Figure 6 shows 2D-WAXD patterns of the TLCP as-spun fibers obtained under the same spinning conditions, as those for the fibers in the SEM images in Figure 5. The patterns confirm that all the as-spun fibers formed domains composed of parallel molecular structures typical of HBA/HNA (73/27 molar ratio) random copolymer [19,20]. The oriented and sharp (110) crystalline peak on the equatorial direction, and the distinct three meridional peaks of m*_n_* with irregular intervals in the 2D-WAXD patterns indicate that a considerable fraction of nematic crystalline structures formed with an orientation direction parallel to the fiber axis [21]. However, the highly concentrated and broad peak near the (110) peak, observed along the equatorial direction, suggests that crystalline structures did not sufficiently develop in the TLCP as-spun fibers, resulting in a considerable fraction of non-crystalline oriented phase in the fibers.

**Figure 7:**
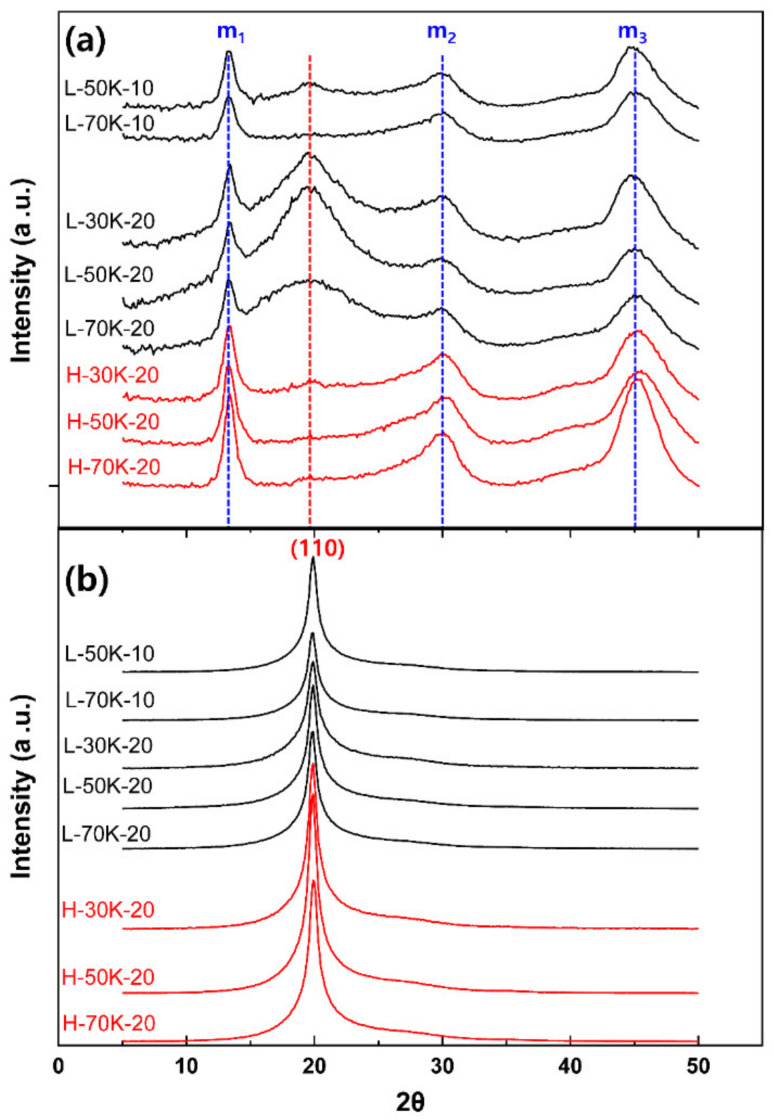
(**a**) Meridional and (**b**) equatorial intensity profiles extracted from the 2D-WAXD patterns of the as-spun TLCP fibers obtained at various spinning conditions.

Figure 7 shows the intensity profiles along the equatorial and meridional directions, which are extracted from the 2D-WAXD patterns. The intensity and position of the (110) peak on the equatorial direction were almost constant for different spinning conditions. The positions of meridional m*_n_* peaks also remained unchanged; however, the peak intensity of all the as-spun fibers obtained at 295 °C tended to be higher than those obtained at 285 °C. 

It is worth noting that another broad peak was observed at 2*θ* ≈ 19.6° on the meridional intensity profiles of all the L-30K/50K/70K-20 as-spun fibers, which were obtained at the high draft of 20 and low spinning temperature of 285 °C. The peak positions of the broad peaks almost corresponded to the Bragg’s angle of the equatorial (110) peak, indicating that a small fraction of non-oriented polycrystalline domains representing the (110) peak exists in the fibers.

**Figure 8:**
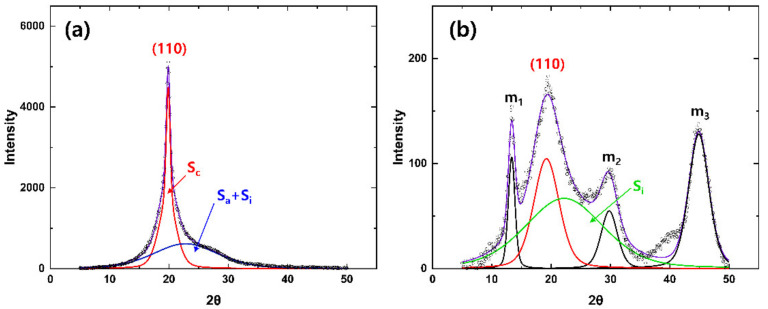
Peak resolution of (**a**) equatorial and (**b**) meridional intensity profiles extracted from the 2D-WAXD patterns of the L-50K-20 as-spun fiber.

To investigate the structural development of the as-spun TLCP fibers according to the spinning conditions, their 2D-WAXD patterns were analyzed in detail. Figure 8a,b shows the peak deconvolution of the equatorial and meridional intensity profiles of the L-50K-20 as-spun fibers using Gaussian–Lorenz fitting [22]. The equatorial intensity profile could be deconvoluted with the sharp crystalline peak of (110) at 2*θ* ≈ 19.8° and the broad peak at 2*θ* ≈ 22.8°, and we supposed that the extracted broad peak was produced by the non-crystalline phase consisting of the highly oriented anisotropic and non-oriented isotropic phases in the as-spun fibers. On the contrary, the meridional profile was well fitted with the three meridional peaks of m_1_, m_2_ and m_3_ at 2*θ* ≈ 13.3, 29.6 and 45.0°, respectively, as well as the non-oriented polycrystalline domains of (110) at 2*θ* ≈ 19.6°, if an additional weak broad peak (S_i_) was assumed at 2*θ* ≈ 22.7°. It is supposed that this additional broad peak was produced due to the non-crystalline isotropic (non-oriented) phase in the fibers, as the peak could be detected in all azimuthal directions, and the peak position almost corresponded with that of the equatorial broad peak of the non-crystalline phase.

Fractions of the crystalline (*X*_c_), non-crystalline anisotropic (oriented) (*X*_a_) and non-crystalline isotropic (non-oriented) (*X*_i_) phases in the TLCP as-spun fibers were calculated with Equations (5)–(7), respectively [23,24].

*X*_c_ = 100 × [*S*_total_ − (*S*_i_ + *S*_a_)]/*S*_total_, (5)

*X*_a_ = 100 × [(*S*_i_ + *S*_a_) − *S*_i_]/*S*_total_, (6)

*X*_i_ = 100 × *S*_i_/*S*_total_, (7)

where *S*_total_ is the total area of the equatorial intensity profile, *S*_a_ is the peak area of the non-crystalline anisotropic phase in the equatorial profile, and *S*_i_ is the peak area of the non-crystalline isotropic phase in the meridional profile. The peak areas of the three m*_n_* peaks and the (110) peak caused by the crystalline phase in the meridional profile were not considered in the calculation of the fractions of the three phases.

The interplanar spacing (*d*-spacing, *d*_110_) of the (110) reflection plane was calculated with Bragg’s Equation (Equation (8)) [23,24].

*d*_110_ = *nλ*/2sinθ, (8)

where *λ* is the X-ray wavelength, and *θ* is the Bragg angle of the peak. The crystallite size for the (110) reflection plane was estimated using Scherrer’s Equation (Equation (9)) [23,24].

*L*_110_ = *Kλ*/*β*_110 _cos*θ*, (9)

where *K* is Scherrer’s constant (=0.9), and *β*_110_ is the intrinsic half-width of the (110) peak.

The crystalline orientation factor (*f*_c_) was estimated from the azimuthal intensity profile of the (110) peak, which were calculated using Equations (10) and (11), respectively [23,24].

*f*_c_ = (180 − *ϕ*)/180, (10)

sin*ϕ* = cos*θ* × sin*X*_E_ (11)

where *ϕ* is the inclination of the c axis to the fiber axis, and *X*_E_ is the half-width of the azimuthal intensity profiles of the (110) peak on the equator.

Table 2 shows the structural data analyzed from the 2D-WAXD patterns of the as-spun TLCP fibers. As expected, overall, all the as-spun fibers displayed high fractions of crystalline (*X*_c_) and non-crystalline anisotropic (*X*_a_) phases, while quite low fractions of the non-crystalline isotropic phase (*X*_i_) were observed. However, it is interesting to note that the L-30K/50K/70K-20 as-spun fibers, which showed a broad non-oriented (110) crystalline peak on the meridional intensity profiles, showed relatively lower crystallinities (*X*_c_) and higher fractions of non-crystalline isotropic phase (*X*_i_) than those of the remaining fibers. In addition, if the meridional m*_n_* peaks were caused by the oriented nematic crystalline structure of typical HBA/HNA (73/27 molar ratio) random copolymer, it is supposed that the area ratio (*S*_m1_/*S*_i_) of the m_1_ peak to the non-crystalline isotropic phase peak in the meridional direction can indirectly represent the volume ratio of the highly oriented crystalline phase to the non-crystalline isotropic phase of the fibers. This ratio tended to increase with the shear rate and the spinning temperature; however, in the case of the L-30K/50K/70K-20 as-spun fibers, the area ratios were significantly lower than those of other as-spun fibers and remained unchanged. These results confirmed that only the L-30K/50K/70K-20 fibers possess highly non-crystalline isotropic phase, which suggests that the different characteristics of structural development, unseen in other TLCP fibers, occurs in the L-30K/50K/70K-20 fibers during the melt spinning process.

The calculated *d*-spacing and lamella size of the (110) plane were similar in all the as-spun fibers, and the crystalline orientation (*f*_c_) showed a tendency to increase as the shear rate, draft and spinning temperature increased.

**Figure 9:**
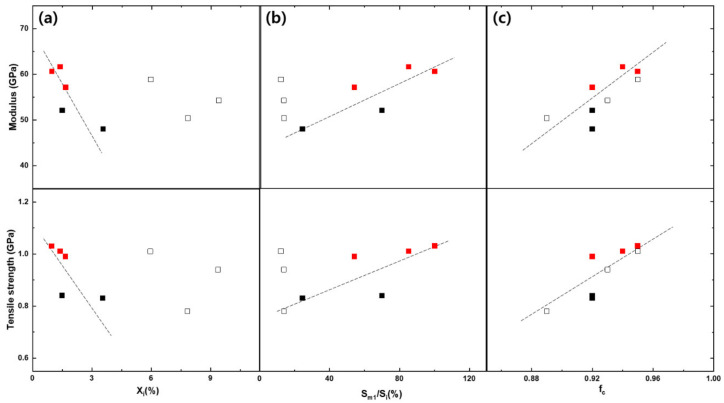
Relationship between the mechanical properties and the structural data ((**a**) the fraction of non-crystalline isotropic phase (*X*_i_), (**b**) area ratio (*S*_m1_/*S*_i_) and (**c**) crystalline orientation factor (*f*_c_)) analyzed from the 2D-WAXD patterns of the as-spun fibers (■: L-50K/70K-10, □: L-30K/50K/70K-20, ■: H-30K/50K/70K-20).

Figure 9 shows the relationship between the mechanical properties of the as-spun TLCP fibers and the structural data calculated from their 2D-WAXD patterns. The tensile strength and modulus in the Figure 9c showed a tendency to increase with the crystalline orientation factor (*f*_c_), indicating that all the as-spun fibers contained a considerable fraction of crystalline phase whose mechanical properties were controlled by the degree of crystal orientation. The mechanical properties also tended to increase as the area ratio (*S*_m1_/*S*_i_) increased (Figure 9b) or decrease as the fraction of non-crystalline isotropic phase (*X*_i_) increased (Figure 9a). Thus, these results mean that the mechanical properties of TLCP as-spun fibers were controlled with the orientation degree of crystalline phase and the total fraction of the highly oriented crystalline phase and non-crystalline anisotropic phase in the fibers.

However, it is worth noting that the L-30K/50K/70K-20 as-spun fibers showed different behavior of mechanical property variation due to the area ratio (*S*_m1_/*S*_i_) and fraction of non-crystalline isotropic phase (*X*_i_) compared with those of the other as-spun fibers. Their positions in the plots deviated from the trend lines (black dotted lines) formed by other as-spun fibers (L-50K/70K-10 and H-30K/50K/70K-20), and their mechanical properties also tended to change independently without being affected by the changes in the area ratio (*S*_m1_/*S*_i_) or *X*_i_ fraction.

Recently, researchers have reported that thermoplastic as-spun fibers obtained at extremely high spinning velocities exhibit a characteristic skin-core structure, wherein the core part shows lower degrees of crystallinity and molecular orientation than the skin [23,24]. Furthermore, this structure has also been reported both in thermotropic [20,25] and lyotropic [26,27] LCP fibers. In the case of thermoplastic fibers, it has been suggested that this skin-core structure can form due to differences in the cooling rates of the skin and core of the fiber in the spin-line [23,24]. Under a high spinning velocity, the skin of the fiber cools faster than the core due to the increased cooling effect on the fiber surface; thus, the temperature-dependent elongational viscosity of the skin increases more than that of the core. Therefore, it can be expected that spinning stress is concentrated in the skin, resulting in rapid molecular orientation and orientation-induced crystallization there. 

To investigate the structure development for a thin filament of cylindrical symmetry in spin-line, simulation studies have been intensively conducted using the steady-state numerical models based on mass balance, momentum balance, energy balance and constitutive equations [18]. Vassilatos et al. [28] suggested the following Equation (12), which applied convective heat transfer coefficients depending on radial velocity element *V_r_* in spin-line heat transfer calculations to predict more accurately the temperature profiles of spin-line, exhibiting significant radial temperature gradients in the cross-section of the fiber.

(12) $$\rho C_{p}\left(V\frac{\partial T}{\partial x}+V_{r}\frac{\partial T}{\partial r}\right)=\frac{k}{r}\frac{\partial }{\partial r}\left(r\frac{\partial T}{\partial r}\right)$$

where *V* and *T* denote the spin-line axial velocity and temperature of the filament at a distance *x* from the nozzle. *r* is radial distance in the filament, and *ρ*, *C_p_* and *k* are the density, specific heat and heat transfer coefficient of the polymer.

**Figure 10:**
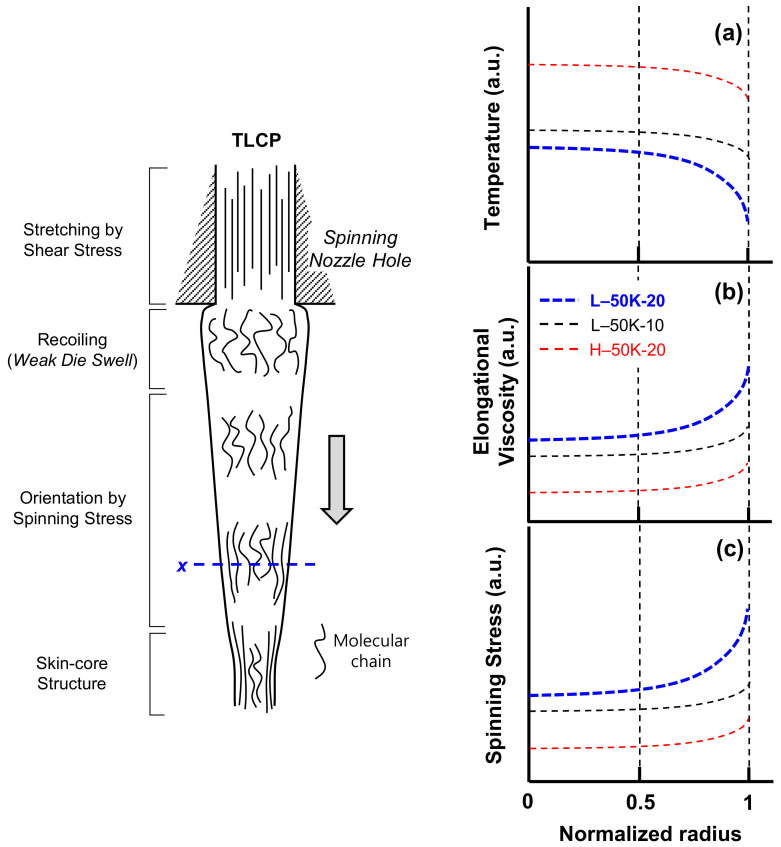
Schematic representation of the structural development of the TLCP fibers in high-speed melt spinning, and the variation of profiles of temperature, elongational viscosity and spinning stress in the cross-section of TLCP fibers (L-50K-20) at the distance of approximately 15 cm from the nozzle in the spinline by the change of the spinning temperature (H-50K-20) and draft (L-50K-10).

Figure 10 shows the variation of profiles of temperature, elongational viscosity and stress in the cross-section of TLCP fibers at the distance of approximately 15 cm from the nozzle in the spin-line by the change in the spinning conditions, calculated with the simulation models [18] and Equation (12). The data of temperature dependence specific heat and elongational viscosity of the TLCP fibers in the spin-line for the calculation were assumed with references [18] and [28,29]. The calculated profiles indicated that if the shear rate in the nozzle hole (i.e., throughput rate and hole diameter) was constant at 50,000 s^−1^, their distributions in the cross-section of the fiber showed a tendency to increase as the draft (i.e., spinning velocity) increased from 10 to 20 and the spinning temperature decreased from 295 to 285 °C. These results are assumed to be attributed to the influence of radial velocity element *V_r_*, increased with the cooling effect at the skin part in the fibers.

Considering these results, we suppose that the heterogeneous structure in the cross-section of the TLCP fibers can be developed due to differences in the cooling rates of the skin and core of the fiber in the spin-line under the specific spinning conditions, and the characteristic results for the L-30K/50K/70K-20 as-spun fibers in Table 2 and Figure 9 were caused by the high distribution of structural development (i.e., skin-core structure) in the fibers. However, there is still a limitation in explain the mechanism of characteristic structure development of the TLCP fibers in spin-line through this practical study, and more systematical simulation study will be conducted to investigate in detail using the on-line measurement of fiber diameter in the future.

On the other hand, the skin-core structure may contribute to the lower mechanical properties of the L-30K/50K/70K-20 as-spun fibers, as shown in Figure 5, because the heterogeneous structure in the cross-section of the fibers can weaken their structural resistance to external stress in the direction of the fiber axis.

## 4. Conclusions

In this study, high-speed melt spinning of HBA/HNA (73/27 molar ratio) TLCP resin was conducted to investigate the characteristic structure development of the fibers under industrial spinning conditions and the correlation between the structural and mechanical properties of the as-spun fibers. The shear thinning behavior of the TLCP resins was still observed in the spinning nozzle hole under the extremely high shear rate of 70,000 s^−1^. The tensile strength and modulus of the fibers increased with the shear rate and draft, and the rate of increase in the mechanical properties due to these two parameters was enhanced at a higher spinning temperature. Two dimensional WAXD patterns of the TLCP as-spun fibers confirmed that all the fibers formed domains comprising parallel-arranged nematic crystalline structure of typical HBA/HNA random copolymer, and a substantial non-crystalline highly oriented structure still existed in the fibers. The mechanical properties of TLCP as-spun fibers increased with the crystalline orientation (*f*_c_) and the total fraction of the highly oriented crystalline and non-crystalline anisotropic phases in the fibers. It is worth noting that the as-spun TLCP fibers, spun at a high draft of 20 and low spinning temperature of 285 °C, formed a skin-core structure in the cross-sections of the fibers, weakening the mechanical properties of the TLCP fibers. Numerical simulation indicated that this structure can be formed by differences in the cooling rates of the skin and core of the fiber in the spin-line. To investigate in detail the mechanism of characteristic structure development of the TLCP fibers in the spin-line, an additional simulation study will be conducted using on-line measurement of fiber diameter in spin-line in the future.

## Figures and Tables

**Table 1:** Sample codes of as-spun TLCP fibers obtained at various spinning conditions.

Sample Code (Spinning Temp. ^1^-SR-Draft)		Throughput Rate (g·min^−1^·hole^−1^)	Shear Rate (×10^3^ s^−1^)	Spinning Velocity (m·min^−1^)	Draft
285 °C	295 °C				
L-30K-15	H-30K-15	0.78	30	550	15
L-30K-20	H-30K-20	0.78	30	750	20
L-30K-25	H-30K-25	0.78	30	950	25
L-50K-10	H-50K-10	1.30	50	650	10
L-50K-15	H-50K-15	1.30	50	950	15
L-50K-20	H-50K-20	1.30	50	1200	20
L-50K-25	H-50K-25	1.30	50	1550	25
L-70K-10	H-70K-10	1.81	70	900	10
L-70K-15	H-70K-15	1.81	70	1300	15
L-70K-20	H-70K-20	1.81	70	1760	20
L-70K-25	H-70K-25	1.81	70	2200	25

^1^ L and H indicate the spinning temperature of 285 and 295 ℃, respectively.

**Table 2:** Characterization data calculated from the 2D-WAXD patterns of the TLCP as-spun fibers obtained at various spinning conditions.

Sample	Fraction ^1^ (%)			*S*_m1_/*S*_i_^2^ (%)	*d*-Spacing (Å)	Lamellar Size (Å) ^3^	*ƒ*_c_^4^
	*X*_c_	*X*_a_	*X*_i_		(*d*_110_)	(*L*_110_)	(110)
L-50K-10	56.0	41.4	3.6	24.6	4.47	57.93	0.92
L-70K-10	55.0	43.2	1.5	70.0	4.48	54.08	0.92
L-30K-20	51.3	42.4	7.8	14.1	4.48	46.34	0.89
L-50K-20	51.7	40.1	9.4	13.9	4.47	55.87	0.93
L-70K-20	53.4	42.6	6.0	12.2	4.48	57.19	0.95
H-30K-20	56.0	41.9	1.7	54.3	4.47	56.52	0.92
H-50K-20	58.7	40.7	1.0	100.2	4.47	58.73	0.95
H-70K-20	58.6	40.6	1.4	85.3	4.46	56.48	0.94

^1^ Fractions of crystalline phase (*X_c_*), non-crystalline oriented anisotropic phase (*X*_a_) and non-crystalline isotropic phase (*X*_i_). ^2^ Area ratio of m_1_ peak (*S*_m1_) and non-crystalline isotropic peak (*S*_i_) in the meridional direction. ^3^ Analyzed by Scherrer’s method. ^4^ Crystalline orientation factor.

## Data Availability

All data generated or analyzed during this study are included in this published article.
